# Three-dimensional imaging of microvasculature in the rat spinal cord following injury

**DOI:** 10.1038/srep12643

**Published:** 2015-07-29

**Authors:** Yong Cao, Tianding Wu, Zhou yuan, Dongzhe Li, Shuangfei Ni, Jianzhong Hu, Hongbin Lu

**Affiliations:** 1 Department of Spine Surgery, Xiangya Hospital, Central South University, Changsha, 410008, China; 2 Department of Sports Medicine, Research Centre of Sports Medicine, Xiangya Hospital, Central South University, Changsha, 410008, China

## Abstract

Research studies on the three-dimensional (3D) morphological alterations of the spinal cord microvasculature after injury provide insight into the pathology of spinal cord injury (SCI). Knowledge in this field has been hampered in the past by imaging technologies that provided only two-dimensional (2D) information on the vascular reactions to trauma. The aim of our study is to investigate the 3D microstructural changes of the rat spinal cord microvasculature on day 1 post-injury using synchrotron radiation micro-tomography (SRμCT). This technology provides high-resolution 3D images of microvasculature in both normal and injured spinal cords, and the smallest vessel detected is approximately 7.4 μm. Moreover, we optimized the 3D vascular visualization with color coding and accurately calculated quantitative changes in vascular architecture after SCI. Compared to the control spinal cord, the damaged spinal cord vessel numbers decreased significantly following injury. Furthermore, the area of injury did not remain concentrated at the epicenter; rather, the signs of damage expanded rostrally and caudally along the spinal cord in 3D. The observed pathological changes were also confirmed by histological tests. These results demonstrate that SRμCT is an effective technology platform for imaging pathological changes in small arteries in neurovascular disease and for evaluating therapeutic interventions.

Acute spinal cord injury (ASCI) is a devastating traumatic event for the central nervous system and is the leading cause of disability, creating a heavy burden on human health worldwide^1^. In physical accidents, violent trauma commonly causes severe damage to the spinal cord and is accompanied by a serious vascular network injury^2^. Although much research has been performed by experts in the field, there is still no effective method to improve the outcome of damaging injuries. Vascular events play an important role in SCI processes, as promotion of neovascularization after injury has the potential to improve neuronal regeneration and long-term functional recovery of the spinal cord^3^,^4^,^5^. Improved understanding of the vascular 3D morphological changes involved in disease development can provide crucial insight into the relationship of these changes with the pathology of SCI, and thus may allow development of novel and effective therapeutic strategies to improve long-term outcomes for patients.

In particular, reports vary regarding the vascular reaction after injury to the spinal cord^6^,^7^. Despite concerted efforts, the 3D morphologic changes in the spinal microvasculature in the SCI process remain a challenging problem that is unsolved, primarily due to the lack of an effective imaging modality.

CT angiography and MR angiography are the most commonly used methods to diagnose the vascular pathology of spinal cord injury in clinical practice. However, the intramedullary artery is located deep within the spinal cord parenchyma, which is outside the limit of detection for these techniques^8^,^9^. The limitations of these clinical techniques arise mainly from an insufficient spatial imaging resolution, which is incapable of detecting a vessel with a diameter of less than 200 μm^10^. Histological sectioning is widely accepted as the gold standard for vessel morphology characterization down to the micrometer scale^11^. However, the sectioning destroys the 3D structural integrity of the spinal cord specimen and provides only two-dimensional (2D) images of the vasculature, whereas the 3D information is much more difficult to obtain. Furthermore, the subsequent preparation step including the staining process is usually time consuming.

Therefore, a high-resolution 3D imaging technique for nondestructive microscopic analysis of the complicated neurovascular networks under conditions of physiologic and pathologic stress is highly desirable. Fortunately, synchrotron radiation micro-computed tomography (SRμCT) imaging techniques have recently been developed and described. SRμCT uses synchrotron radiation beam light, which has evolved as an increasingly useful technique for characterizing the 3D structures of samples in multiple biomedical applications^12^.

The high intensity, high coherence, and monochromatic characteristics of SR beam light have enabled the SR-based imaging technique to obtain high-resolution images of vessel networks at micro- and even submicro-levels. In experimental studies, this method has been applied to the visualization of the 3D morphology of the microvasculature in the heart, hind limbs, tumors, and the brain^13^,^14^,^15^,^16^,^17^. However, the method’s utility for 3D visualization of the pathologic changes of angioarchitecture in trauma-induced spinal cords has not been investigated.

In the present study, this tool has been applied to investigate the 3D morphology changes in the spinal cord microvasculature under both normal and post-traumatic conditions. All of the experiments were performed using the X-ray imaging and biomedical application beamline (BL13W1) at the Shanghai Light Source in China, the third-generation Shanghai Synchrotron Radiation Facility (SSRF). A schematic depiction of the experimental setup is shown in Fig. 1. The goal of our study was to demonstrate the feasibility of the 3D visualization of neurovascular structure by SRμCT and to investigate the potential vascular pathology following SCI.

## Results

### Imaging efficiency

**Figure 2:**
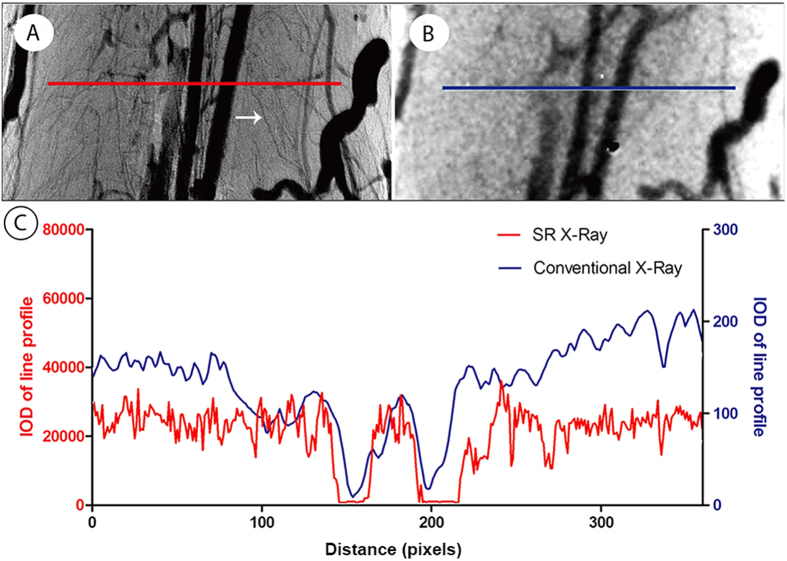
Comparison of initial projected images between synchrotron radiation (SR) and conventional X-ray imaging. (**A**) Projected image by synchrotron radiation imaging. (**B**) Projected image by ordinary X-ray imaging. (**C**) Representation of the intensity distribution of the line profiles located in the correspondence images in **B** and **C** indicating that the SR (red line) has a greater capacity to distinguish the microvessels.

To compare imaging efficiencies, the same sample was subjected to synchrotron radiation and conventional X-ray imaging successively. Two typical X-ray projected images are compared in Fig. 2. In contrast to the conventional X-ray imaging result, the synchrotron radiation image provided a legible background projection, in which many small vessels filled with contrast agent were easily differentiated (Fig. 2A). Conversely, it was difficult to distinguish the small vessels and tissue background in the conventional X-ray projection due to the weak resolution (Fig. 2B). A tiny vessel of approximately 7.4 μm in diameter is highlighted with an arrow in the synchrotron radiation image, which failed to appear at all in the conventional X-ray projected image (Fig. 2A, white arrow). The integral optical density (IOD) representing the grayscale distribution crossing the line profile of the same section is shown in Fig. 2C. Both the red and blue IOD distribution curves appeared as parallel waves in accordance with the intensity arrangement of imaging vessels. As expected, the wave vibration of the red synchrotron radiation curve is more frequent than that of the blue line profile in the conventional X-ray image. It was not surprising that the synchrotron radiation image displayed a superior level of detail, with more small blood vessels apparent within the angiography, compared with conventional X-ray imaging.

### 3D imaging of spinal cord vascular morphology

**Figure 3:**
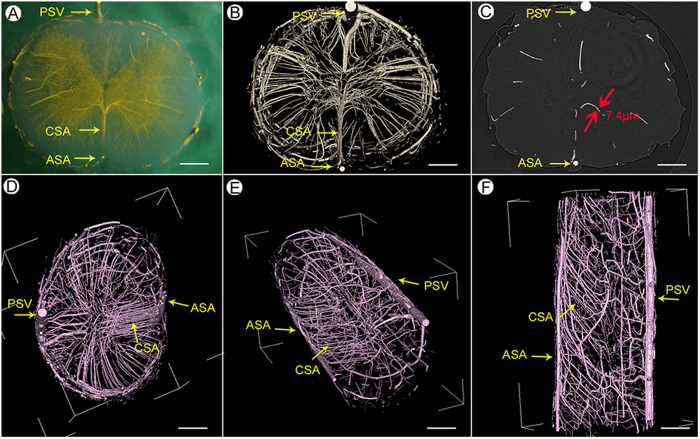
3D image of spinal cord vascular morphology. (**A**) The stereomicroscope image of the spinal cord. (**B**) SRμCT 3D image of spinal cord microvasculature. (**C**) Linear length measurements of vessels in the gray matter region of the spinal cord down to 7.4 μm. (**D**–**F**) An intact vasculature with pink pseudo-color to allow the viewing of different levels of vascular detail at the top (**D**), oblique (**E**), and sagittal (**F**) orientation. (PSV = posterior spinal vein; ASA = anterior spinal artery; CSA = central sulcal artery.) Bar = 200 μm.

We adopted an approach to visually render the 3D morphology of the rat spinal cord microvasculature. The stereomicroscopic images of the transverse sections indicate that the vessels were filled with the yellow contrast agent and the butterfly shape of the blood supply to the gray matter of the spinal cord is clearly visualized (Fig. 3A). The 3D vascular framework of the spinal cord is shown in Fig. 3B. The main blood supply, including the central sulcus arterial system and the peripheral artery system of the spinal cord, was detected, and the smallest vessel detected appeared to be approximately 7.4 μm in diameter (Fig. 3C).

An intact, pinkish vasculature can be seen in the multi-perspective and multi-angle images (Fig. 3D–F). The vessels with different diameters spontaneously form an orderly branching and natural weaving structure. These images show that the central sulcal artery (CSA), the most important source of blood to the spinal cord, has branches to the inner parenchymal parts at certain angles. Furthermore, the dense intramedullary component of the spinal cord can be visualized via the 3D representation. The concentrated distribution in the region of the grey matter presumably indicates that neuronal cell bodies are highly dependent on nutrients and oxygen supply compared with the nerve fibers of the white matter.

### 3D Image optimization and blood supply arrangement

**Figure 4:**
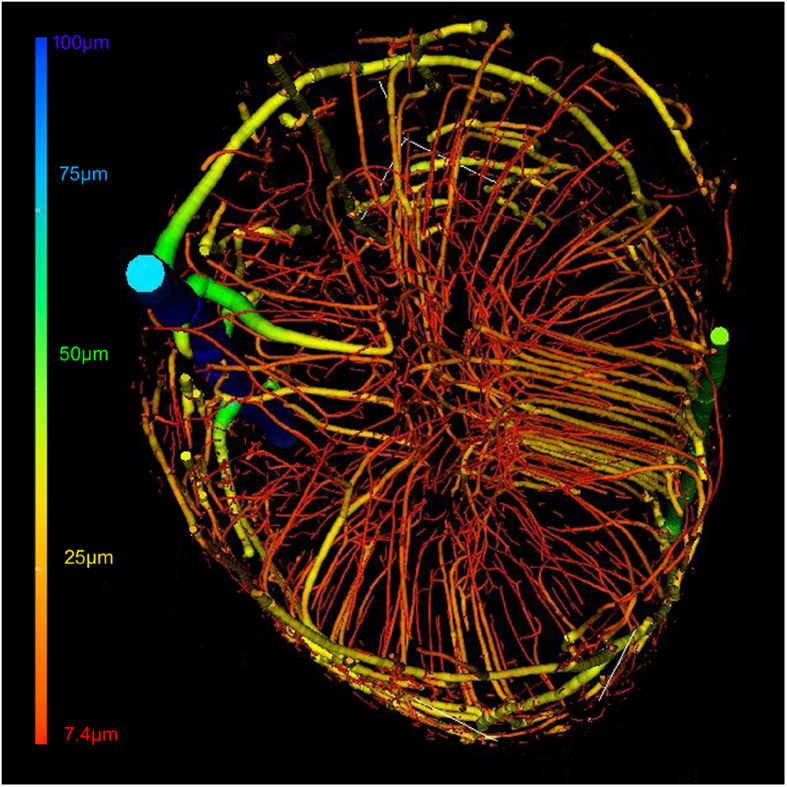
3D pseudo-colored microvasculature of the spinal cord. The color bar indicates color associations with the different vessel diameters.

After segmentation, the vessels with different diameters could be defined using the color coding to represent the vessel thickness. The intact vasculature with a pseudo-pink color is visualized from the top in Fig. 4. In this way, the distribution of normal blood vessel can be clearly observed and provides a better pictorial representation of the unique spinal cord vasculature. The various vessels nourishing the spinal cord ranged from 7.4 μm to 100 μm in diameter (Fig. 4).

**Figure 5:**
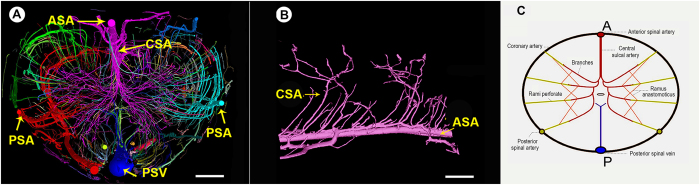
Synchrotron radiation image optimization. (**A**) The pseudo-color images of whole 3D volumetric rendering of the intact vasculature of the normal spinal cord, painted to display the different regions from the top view. (**B**) The characteristic morphology of the CSA. (**C**) A schematic depiction of the normal blood supply pattern of the rat spinal cord. (A =anterior; P = posterior.) Bar = 200 μm.

To analyze the vascular arrangement of the blood supply network in the spinal cord, several pseudo colors were partitioned to display the distinct regions (Fig. 5A). The main nutrient vessels, including the anterior spinal artery (ASA), posterior spinal artery (PSA), central sulcal artery (CSA) and posterior spinal vein (PSV), are distinguishable. To define the characteristic morphology of the CSA, the vasculature was partitioned and separated from the whole 3D image. The sixth branch level of these arteries can be visualized and viewed in a multi-perspective image (Fig. 5B).

The intramedullary blood supply arrangement of the spinal cord is illustrated in Fig. 5C. In accordance with previous descriptions^18^, the central sulcal artery derives from the anterior spinal artery and bilaterally branches into the gray matter. The rami perforantes originated from the posterior spinal arteries and pial arterial plexus, vertically providing blood to intrinsic tissues. We also observe that most of the tiny rami anastomotici are arranged to connect the central sulcal artery system with the rami perforantes system and inosculate into a complicated blood supply feeding the spinal cord.

### 3D vascular morphology changes after injury

**Figure 6:**
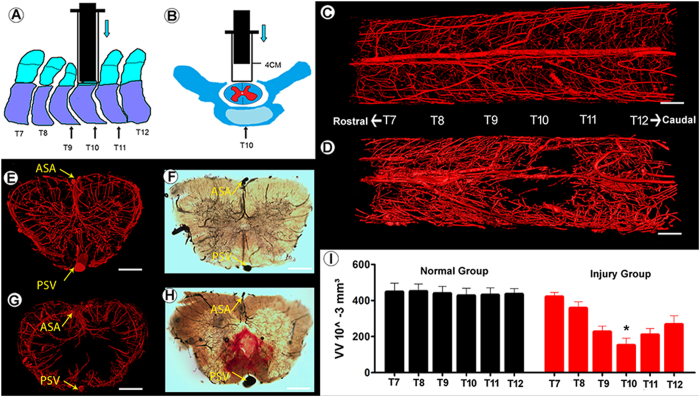
3D morphology changes of the rat spinal cord microvasculature 1 day post-injury. (**A,B**) Depiction of the spinal cord injury process. The injured epicenter was located at the T10 level. The intact view of 3D microvasculature of the normal (**C**) and injured (**D**) spinal cord. The transection view of the T10 level of the spinal cord microvasculature before (**E**) and after (**G**) injury obtained by SRμCT. The correspondence image of the normal (**F**) and injured (**H**) spinal cord vasculature detected by optical microscope. (**I**) The VV of normal and injured spinal cord calculated from the indicated level of the image (**C**) and (**D**) demonstrated that the VV at the T10 level (black star) decreases significantly.

Figure 6A,B depict the spinal cord injury process. The injured epicenter is located at the 10th level thoracic vertebra. The normal 3D image of the rat spinal cord microvasculature is shown in Fig. 6C. To characterize the architectural alterations in vascular morphopathology, typical images of traumatized rat spinal cords are depicted in Fig. 6D. The injury epicenter emerged as an expanded avascular cavity. The local blood supply was severely disordered 1 day after an acute injury. In the transection view, although the large extramedullary vessels (anterior spinal artery, posterior spinal vein) and vasculature in the white matter remain, many tiny blood vessels located in the gray matter are severely disrupted (Fig. 6E,G). An avascular area was observed in the gray matter of the injured cord segment. Given the dysfunction of blood flow, a posttraumatic decrease of the local blood supply volume was inevitable. These pathological changes to the vessels were confirmed by histological testing (Fig. 6F,H). When comparing the VV with the normal spinal cord, we found that the VV in the injured cord epicenter (T10^th^ level) was significantly decreased (Fig. 6I).

**Figure 7:**
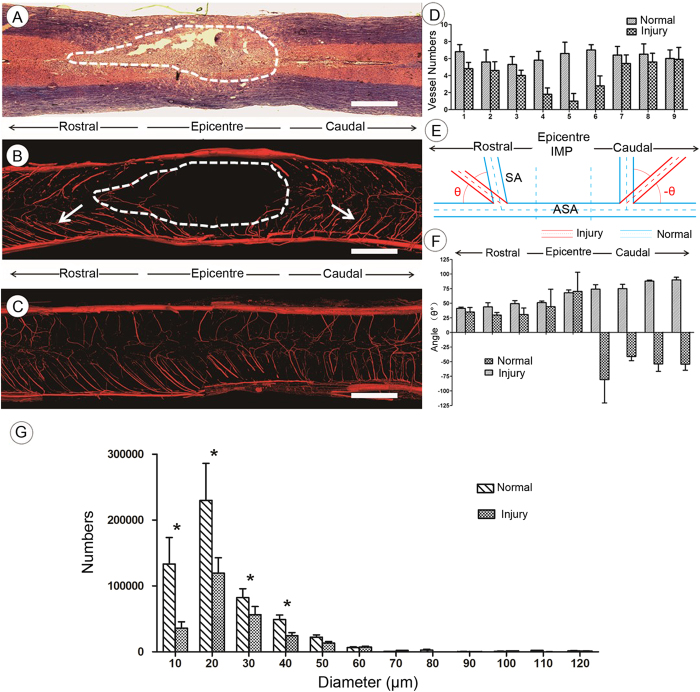
The characteristic morphology changes of the CSA 1 day after SCI. (**A**) HE staining. (**B**) 3D image of CSA demonstrating the collapsed bent vessels (arrow) and diminished perfusion (dotted circle). (**C**) 3D CSA image from normal spinal cord. (**D**) The quantitative analysis of CSA numbers decrease in the injury epicenter after SCI. (**E**,**F**) Depiction of the vessel collapse pathology. CSA has fallen toward both rostral and caudal orientations with different θ° alterations due to internal IMP elevation. (**G**) The vessel diameter distribution of the spinal cord before and after spinal cord injury. Bar = 500 μm.

HE staining demonstrated profound cord damage 1 day post-injury both within and beyond the epicenter (Fig. 7A). The morphopathological characteristics of the injured angioarchitecture of the CSA are highlighted in Fig. 7B. Under normal conditions, the CSA derives from the anterior spinal artery and branches into the gray matter at a regular angle (Fig. 7C). Following trauma, the injury was not concentrated at the epicenter but instead expanded rostrally and caudally along the spinal cord in 3D to form a characteristic long spindle shape (Fig. 7B). Meanwhile, the CSA numbers in the injury epicenter were significantly decreased relative to those in uninjured rats (Fig. 7D). The CSAs are additionally pathologically distorted toward both a rostral and caudal orientation (Fig. 7B, arrow). We inferred that the acute aggravation of the intramedullary pressure (IMP) at the injury epicenter, and along the extended rostral and caudal directions, is a cause of the bilateral displacement of spinal vasculature. To illustrate the vessel displacement pathology, the vascular morphology changes are shown in Fig. 7E. The intersection angle (θ) between CSA and ASA was defined as positive, as the angle was no more than 90°. Calculation of the normal average degree of θ gave a value of 68° ± 3.6°. Following trauma, however, the right CSA falls back toward the caudal orientation with a negative −θ degree, and the left CSA was rostrally displaced with an average θ degree of 49° ± 8.7° (Fig. 7F). The alteration in θ degree indicates an abnormal dislocation and drift of vasculature in the spinal cord after injury. To gain further insight into the vessel morphometry associated with the normal and injured spinal cord vasculature, we calculated the vessel diameters and found that large numbers of blood vessels were lost after SCI, and most of those remaining were less than 30 μm in diameter after (Fig. 7G).

Through statistical analysis, the VV was also significantly reduced 1 day after SCI (P < 0.01). To demonstrate the angioarchitecture, distinctive parameters of the vascular morphology at the 3D level were selected for quantification of the vasculature in greater detail. The VN decreased significantly after SCI (P < 0.01), and the vessel node number (VNN) also significantly decreased after SCI (P < 0.01). The quantitative data determined for specimens in the present study are listed in Table 1.

## Discussion

Clear identification of the vascular reaction to SCI will enable a better understanding of the associated pathological changes, ultimately driving the development of novel strategies for therapeutic interventions. Although studies have been performed to investigate this system, the detailed mechanisms underlying this process have not been completely elucidated.

In the present study, we successfully established a procedure that allows 3D visualization of spinal cord microvasculature, and we provide new insight into the vascular reactions to spinal cord trauma. Furthermore, the 3D rendering of the spinal cord microvasculature could be clearly visualized from different perspectives, even for vessels with diameters as small as approximately 7.4 μm, which approaches the capillary scale. Utilizing the vessel tree analysis method^19^, visualization of the vascular network was optimized by color-coding to provide a better understanding of the unique character of spinal cord vasculature post-injury, which was not previously possible using classical histological methods. Additionally, to trace vessel reaction and regeneration after injury, this method provides a better way to visualize 3D images of the vessel without sectioning. The overlapping vessel structures found in 2D images are also not an accurate way to quantify the vascular morphology parameters, because only partial information of the vasculature is provided.

Micro-CT presents unique opportunities for high-resolution 3D imaging of microvasculature in place of microscopy examination that depends on histological sectioning^20^. The spatial resolution of micro-CT strictly relies on the X-ray source, which is determined by the type of apparatus used. Currently, the desk micro-CT has a resolution of approximately 20 μm, as shown in previous studies^21^,^22^. It has been difficult to achieve sufficient spatial resolution and luminous flux to differentiate a tiny vasculature among the parenchyma of the spinal cord. For imaging superiority and comparative quantification of vascular alterations of traumatized spinal cords, this study visualized the neurovascular network structure by the novel micro-imaging methodology, SRμCT. Our results show that reconstructed SRμCT images facilitate 3D representations of angioarchitectural features in the microvascular atlas of rat models of spinal cord injury.

The excellent imaging properties of a synchrotron radiation light source are attributable to its highly coherent, parallel, and brilliant monochromatic X-ray beam. Owing to the imaging superiority offered, synchrotron radiation has previously been used to investigate the microvascular changes in animal models of brain stroke and tumor neoangiogenesis^23^,^24^. The resolution limitation appeared to be as small as ~2.0 μm, which is close to the capillary level and is suitable for neovascularization exploration^25^. Herein, our results first demonstrated the normal and posttraumatic spinal vasculature in 3D. The method provided a micro-dimensional level of detection and quantification of angioarchitecture changes of the blood vessels deep within the spinal cord in rat SCI models. We predict that SRμCT will be a promising tool for exploring the 3D neurovascular morphological anatomy and evaluating the morphopathological features of animal models of microvessel-related diseases.

Our results show that the communicating blood flow pattern of the CSA and rami perforantes systems nourishes the deep neural parenchyma of the gray matter and has a high microvessel density, indicating that the neurons in this area have more metabolic requirements than those in white matter. The unique anatomical and functional properties of the blood supply to the spinal cord determine the susceptibility to blood loss and oxygen deficiency in cell bodies as compared with the pyramidal tracts.

Spinal cord injury is a devastating event accompanied by vascular destruction and neurological deficits. Previous studies have delineated the source of the blood supply to the spinal cord and indicate the close relationship between neural recovery and microcirculatory improvement^3^,^4^,^5^,^6^,^7^. Our results present a high-resolution 3D view of morphopathological alterations to the spinal cord vasculature post-injury, which have not previously been reported. The spinal cord was found to be susceptible in its response to primary trauma. The segmented vessel network showed severe structural damage and volumetric recession. The pathological vasculature led to the aggravation of local malignant microcirculation and secondary spinal cord injury^18^. In addition, vascular deformation was confirmed by the characteristic morphology of the CSA disruption both caudally and rostrally. The vascular vulnerability in response to mechanical insult was easily deduced. Although the instant trauma caused the physical distortion of cord vasculature, the rapidly elevated intramedullary pressure (IMP) appeared to be another important factor resulting in vascular collapse. Thus, it is important to note that the blood supply to the spinal cord is required to protect the residual neural parenchyma from further injury.

The principle of the SRμCT is based on the attenuation of X-rays passing through the sample. Unlike that of bones, visualization of blood vessels relies on vascular contrast perfusion^26^,^27^. In the present study, to obtain a successful perfusion, we measured the arterial pressure and perfused contrast at a reasonable pressure not exceeding physiologic values, as previously described^22^. The angiography was sufficient for quantitative determination of the vessel network. However, the major limitation of this research is the lack of time-lapse *in vivo* imaging at the microlevel, which would allow dynamic 3D monitoring of changes after spinal cord injury without sacrificing a rat for each imaging session. Because the spine, unlike the cranium, is close to the heart and lungs, the motion artifacts cause by the heart beat preclude reconstruction of a 3D image of the spinal cord microvasculature. Thus, further development of SRμCT from pre-clinical to clinical applications for vascular disease diagnosis will require more effective conditions for *in vivo* imaging.

In conclusion, our study shows that SRμCT provides high-resolution 3D images of vascular architectural physiopathology in normal and injured spinal cord segments. Using this analytical technique in rat models, we investigated trauma-induced pathological changes to blood vessels via redefined quantitative metrics and spatial orientation. The imaging and analysis technique provided an effective technology platform for imaging pathological changes in neurovascular disease and for evaluating the efficacy and safety of blood-related drugs in microvasculature repair and regeneration research.

## Methods and Materials

### Experimental animals and Ethics statement

All experiments were conducted in strict compliance with the guidelines established by the Animal Care and Use Committee of the Central South University. The protocol was approved by the Committee on the Ethics of Animal Experiments of Central South University (Permit Number: 201303243). A total of 10 Sprague-Dawley (SD) male rats (250–300 g) were randomly divided into a normal control group (n = 5) and an SCI group (n = 5).

### Construction of an experimental SCI model

Five rats in the SCI group were induced by a modified Allen’s weight-drop apparatus as described previously^28^. Briefly, the rats were anesthetized with an intraperitoneal injection (i.p.) of 10% chloral hydrate (3 ml/kg). The incision was located on the dorsomedian line centered at the T10 vertebra. A laminectomy was performed to expose the spinal cord at this level. The exposed spinal cord segment, approximately 4 mm long, was subjected to a moderate vertical impacting load. An immediate intramedullary hemorrhage was observed, the rat tail swung pendulously, and both hind limbs retracted convulsively after the impact, signaling that the SCI rat model was successfully produced. A group of five normal rats that were subjected only to laminectomy served as the normal control group.

All the surgery was performed in a constant environment. After surgery, the rats were housed in cages and given free access to food and water.

### Sample preparation

One day post-injury, all rats in both the normal control group and the SCI group were anesthetized with 10% chloral hydrate (3 ml/kg, i.p.). A thoracotomy was rapidly performed to expose the heart. Heparinized saline was rapidly infused into the circulatory system via the ascending aorta, allowing an effective drain of blood flow. Then, 10% buffered formalin was perfused for tissue fixation. A proportional mixture of contrast agents (Microfil MV-122, Flow Tech, CA, USA), described previously^29^, was infused into the ascending aorta via a perfusion pump with a filling rate of 0.5 mL/min at 140 mmHg for 5 min. Thereafter, all animals were preserved at 4 °C to cure overnight. The next day, the T10 thoracic cord segment with a length of 4 mm was harvested and fixed in a 10% buffered formalin solution. Twenty-four hours later, all specimens were dehydrated with a gradient of ethyl alcohol for 12 hours and then prepared for SRμCT scanning.

### SRμCT setup and image acquisition

SRμCT scanning was performed at the BL13W1 biomedical beamline in the Shanghai Synchrotron Radiation Facility (SSRF) in China. The SSRF is a third-generation synchrotron radiation light source, with a 3.5 GeV electron beam energy storage ring and a 180 mA beam current. This beamline can provide tunable photon energy that ranges from 8 to 72.5 keV. The synchrotron radiation beam was monochromatized by a double-crystal monochromator. The sample was fixed on the rotary stage, which was exposed to the SR light path. Subsequently, the SR light transmitted through the object was detected by the CCD camera with 3.7 × 3.7 μm/pixels (Photonic Science, UK). To obtain high X-ray attenuation contrast images, the monochromatic X-ray energy was adjusted to 15 keV, the exposure time was set to 2.5 s, and the sample-to-detector distance (SDD) was adjusted to 2 cm. While the sample stage rotated 180°, a total of 720 initial projecting images were captured. In addition, 12 flat-field images were recorded without sample in the SR beamline path. Five dark-field images were captured when the light source was switched off; they were used to subtract background signal and normalize the image intensity.

After SRμCT scanning, all initial projection images were transformed into digital slice sections using the GPU-CT-Reconstruction software (applied by the BL13W1 experimental station) based on the filtered back projection (FBP) algorithm. A series of slices was reconstructed using the VG Studio Max 3D reconstruction software (version 2.1, Volume Graphics GmbH, Germany) to render the 3D images.

### Vasculature image analysis

To evaluate the local differences in the vessel parameters of the normal and injured spinal cord, two regions of interest (ROI) at the same level of the spinal cord (approximately 4 mm) were selected in each tomography dataset.

### Vessel segmentation

After scanning, the 3D image of each ROI was imported into Image J software (W.S. Rasband, National Institutes of Health, Bethesda, Maryland, USA) for blood vessel extraction and calculation of vascular morphological parameters. The vasculature was segmented from the tissue using an iterative gray level-based threshold algorithm^30^. A binary 3D vascular structure was obtained. To eliminate small, disconnected objects and gaps in the vasculature, the binary image was subjected to a 3D size filter to remove isolated background and foreground regions smaller than three voxels.

### Characterization of vascular morphology

**Figure 8:**
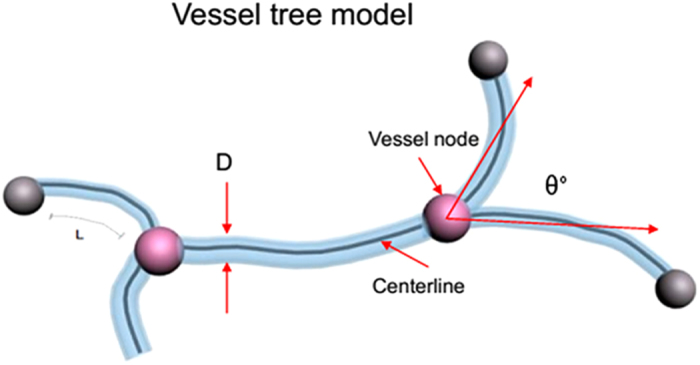
Schematic showing the vessel tree model used for quantification of the spinal cord microvasculature network. θ° was the bifurcation angle of the vessel. D: 2 vessel radii. L: vessel number between two vessel nodes.

After segmentation, several morphometric parameters of the vasculature were computed to characterize the 3D vascular architecture. The vascular volume (VV) of the ROI was determined by computing the fractional occupancy of the binary vascular structure within each ROI. The Image J 3D Skeletonization plugin was used to automatically extract the vascular centerlines or 3D skeleton^31^. The vessel numbers (VN)—the sum of the numbers in label L in Fig. 8—represent vessels with the same diameters between two bifurcation points. The vessel node number (VNN) was computed by summing all bifurcation points. To measure the vessel radius, a 3D Euclidean distance map (EDM)^32^ of the vasculature was computed using the ChamferMap module in Amira (Mercury, Richmond, TX, USA). This EDM represents the smallest distance between each vessel voxel and the background. Vessel radius was determined by multiplying the binary skeletonized image by the EDM. Figure 8 illustrates these parameters. The vessel tree model was used to quantify the spinal cord microvasculature network. The VNN, vessel diameter (VD) (mean of 2 radii), VN, and VV were calculated and compared between the SCI and control groups.

### Histomorphological observation

To confirm the imaging value of SRμCT, the spinal cord samples were photographed using a stereomicroscope (SZX12, Olympus America, Melville, NY) after scanning. They were then sectioned using a double-edged razor blade into transverse slices with thicknesses of approximately 300 μm for morphological observation of the intra-medullary vessels. The corresponding sample mentioned above was immersed in methyl salicylate. When the tissues became transparent, they were photographed with an optical microscope (Leica DM4000B, Germany), and the photographs were compared with the 3D images obtained by SRμCT.

### Data analysis

Statistical analysis was performed using the SPSS software package, version 17.0 (University of Cambridge, Cambridge, UK). All data are presented as the mean ± SD. The vascular morphology parameters measured by the SRμCT results of normal and injured groups were analyzed using Student’s t-test, and a p-value < 0.05 was considered statistically significant.

## Additional Information

**How to cite this article**: Cao, Y. *et al*. Three-dimensional imaging of microvasculature in the rat spinal cord following injury. *Sci. Rep*. **5**, 12643; doi: 10.1038/srep12643 (2015).

## Figures and Tables

**Figure 1:**
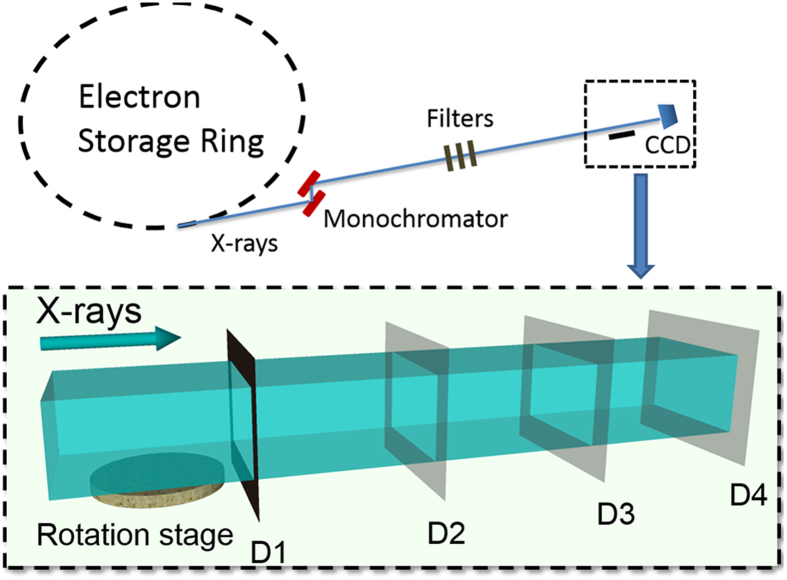
Schematic depiction of the experimental apparatus at the BL13W1 beamline experimental station in SSRF. The distance between the sample and detector enables absorption and phase-contrast imaging. (Absorption contrast imaging: D1; phase-contrast imaging: D2, D3, D4.)

**Table 1:** The 3D morphometric parameters of vasculature in ROI selected from the rat spinal cord.

Parameters	Normal	Injury
Vessel Volume (mm^3^)*	0.84 ± 0.25	0.64 ± 0.44
Vessel Number/μm^3^*	426 ± 140	303.75 ± 39.65
Vessel Node Number/μm^3^*	69.7 ± 14.2	40.1 ± 15.6

^*^P < 0.01

## References

[b1] Wyndaele M. & Wyndaele J. J. Incidence, prevalence and epidemiology of spinal cord injury: what learns a worldwide literature survey? Spinal Cord 44, 523–529 (2006). 16389270 10.1038/sj.sc.3101893

[b2] Figley S. A., Khosravi R., Legasto J. M., Tseng Y. F. & Fehlings M. G. Characterization of Vascular Disruption and Blood–Spinal Cord Barrier Permeability following Traumatic Spinal Cord Injury. J Neurotrauma 31, 541–52 (2014). 24237182 10.1089/neu.2013.3034 PMC3949504

[b3] Liu Y. . An engineered transcription factor which activates VEGF-A enhances recovery afterspinal cord injury. Neurobiol Dis 37, 384–93 (2010). 19879362 10.1016/j.nbd.2009.10.018

[b4] Lutton C. . Combined VEGF and PDGF treatment reduces secondary degeneration after spinal cord injury. J Neurotrauma 29, 957–70 (2012). 21568693 10.1089/neu.2010.1423

[b5] Widenfalk J. . Vascular endothelial growth factor improves functional outcome and decreases secondary degeneration in experimental spinal cord contusion injury. Neuroscience 120, 951–60 (2003). 12927201 10.1016/s0306-4522(03)00399-3

[b6] Loy D. N. . Temporal progression of angiogenesis and basal lamina deposition after contusive spinal cord injury in the adult rat. J Comp Neurol 445, 308–24 (2002). 11920709 10.1002/cne.10168

[b7] Mautes A. E., Weinzierl M. R., Donovan F. & Noble L. J. Vascular Events After Spinal Cord Injury: Contribution to Secondary Pathogenesis. Phys Ther 80, 673–87 (2000). 10869130

[b8] Vargas M. I. . Dynamic MR angiography (MRA) of spinal vascular diseases at 3T. Eur Radiol 20, 2491–5 (2010). 20473612 10.1007/s00330-010-1815-6

[b9] Yoshioka K. . MR Angiography and CT Angiography of the Artery of Adamkiewicz: Noninvasive Preoperative Assessment of Thoracoabdominal Aortic Aneurysm. Radiographics 23, 1215–25 (2003). 12975511 10.1148/rg.235025031

[b10] Mori H. . Visualization of penetrating transmural arteries *in situ* by monochromatic synchrotron radiation. Circulation 89, 863–71 (1994). 8313576 10.1161/01.cir.89.2.863

[b11] Ertürk A. . Three-dimensional imaging of solvent-cleared organs using 3DISCO. Nat Protoc 7, 1983–95 (2012). 23060243 10.1038/nprot.2012.119

[b12] Suortti P. & Thomlinson W. Medical applications of synchrotron radiation. Phys Med Biol 48, R1–35 (2003). 12884920 10.1088/0031-9155/48/13/201

[b13] Shirai M. . Synchrotron Radiation Imaging for Advancing Our Understanding of Cardiovascular Function. Circ Res 112, 209–21 (2013). 23287456 10.1161/CIRCRESAHA.111.300096

[b14] Zhang M. Q. . Three-dimensional visualization of rat brain microvasculature following permanent focal ischaemia by synchrotron radiation. Br J Radiol 87, 20130670 (2014). 24702152 10.1259/bjr.20130670 PMC4075551

[b15] Schültke E. . Dual energy CT at the synchrotron: A piglet model for neurovascular research. Eur J Radiol 79, 323–7 (2011). 20732772 10.1016/j.ejrad.2010.07.002

[b16] Lu W. . Detection of Microvasculature in Rat Hind Limb Using Synchrotron Radiation. J Surg Res 164, e193–9 (2010). 20828725 10.1016/j.jss.2010.05.015

[b17] Lundström U. . X-ray phase contrast with injected gas for tumor microangiography. Phys Med Biol 59, 2801–11 (2014). 24801363 10.1088/0031-9155/59/11/2801

[b18] Martirosyan N. L. . Blood supply and vascular reactivity of the spinal cord under normal and pathological conditions. J Neurosurg Spine 15, 238–51 (2011). 21663407 10.3171/2011.4.SPINE10543

[b19] Lang S. . Three-dimensional quantification of capillary networks in healthy and cancerous tissues of two mice. Microvasc Res 84, 314–22 (2012). 22796313 10.1016/j.mvr.2012.07.002

[b20] Dunmore-Buyze P. J. . Three-dimensional imaging of the mouse heart and vasculature using micro-CT and whole-body perfusion of iodine or phosphotungstic acid. Contrast Media Mol Imaging 9, 383–90 (2014). 24764151 10.1002/cmmi.1588

[b21] Long H. Q. . Value of micro-CT for monitoring spinal microvascular changes after chronic spinal cord compression. Int J Mol Sci 15, 12061–73 (2014). 25003643 10.3390/ijms150712061 PMC4139829

[b22] Hu J. Z. . Three-dimensional alteration of microvasculature in a rat model of traumatic spinal cord injury. J Neurosci Methods 204, 150–8 (2012). 22101144 10.1016/j.jneumeth.2011.10.018

[b23] Lu H. . Netrin-1 hyperexpression in mouse brain promotes angiogenesis and long-term neurological recovery after transient focal ischemia. Stroke 43, 838–43 (2012). 22223243 10.1161/STROKEAHA.111.635235

[b24] Pabst A. M. . Imaging angiogenesis: perspectives and opportunities in tumour research-a method display. J Craniomaxillofac Surg 42, 915–23 (2014). 24518362 10.1016/j.jcms.2014.01.010

[b25] Stolz E. . Angioarchitectural changes in subacute cerebral venous thrombosis. A synchrotron-based micro- and nano-CT study. Neuroimage 54, 1881–6 (2011). 20974267 10.1016/j.neuroimage.2010.10.056

[b26] Grabherr S. . Angiofil-mediated visualization of the vascular system by microcomputed tomography: A feasibility study. Microsc Res Tech 71, 551–6 (2008). 18393302 10.1002/jemt.20585

[b27] Vasquez S. X. . Optimization of microCT imaging and blood vessel diameter quantitation of preclinical specimen vasculature with radiopaque polymer injection medium. PLos One 6, e19099 (2011). 21533123 10.1371/journal.pone.0019099 PMC3078938

[b28] Koyanagi I., Tator C. H. & Lea P. J. Three-dimensional analysis of the vascular system in the rat spinal cord with scanning electron microscopy of vascular corrosion casts. Part 2: Acute spinal cord injury. Neurosurgery 33, 285–292 (1993). 8367052

[b29] Ghanavati S., Yu L. X., Lerch J. P. & Sled J. G. A perfusion procedure for imaging of the mouse cerebral vasculature by X-ray micro-CT. J Neurosci Methods 221, 70–7 (2014). 24056228 10.1016/j.jneumeth.2013.09.002

[b30] Reichold J. . Vascular graph model to simulate the cerebral blood flow in realistic vascular networks. J Cereb Blood Flow Metab 29, 1429–1443 (2009). 19436317 10.1038/jcbfm.2009.58

[b31] Lee T. C., Kashyap R. L. & Chu C. N. Building skeleton models via 3-D medial surface/axis thinning algorithms. Graph Models Image Processing 56, 462–478 (1994).

[b32] Tsai P. S. . Correlations of neuronal and microvascular densities in murine cortex revealed by direct counting and colocalization of nuclei and vessels. J Neurosci 29, 14553–14570 (2009). 19923289 10.1523/JNEUROSCI.3287-09.2009 PMC4972024

